# Anti-GABAB receptor encephalitis: clinical and laboratory characteristics, imaging, treatments and prognosis

**DOI:** 10.3389/fimmu.2024.1442733

**Published:** 2024-10-09

**Authors:** Dongrui Li, Shenghua Zong, Yaobing Yao, Peter C. Molenaar, Jan G. M. C. Damoiseaux, Hui Li, Rob P. W. Rouhl, Pilar Martinez-Martinez

**Affiliations:** ^1^ Department of Neurology, The First Affiliated Hospital of Zhengzhou University, Zhengzhou, China; ^2^ Department of Psychiatry and Neuropsychology, School for Mental Health and Neuroscience (MHeNS), Maastricht University, Maastricht, Netherlands; ^3^ Neuroimmunology Group, KingMed Diagnostic Laboratory, Guangzhou, China; ^4^ Central Diagnostic Laboratory, Maastricht University Medical Center (MUMC)+, Maastricht, Netherlands; ^5^ Department of Respiratory Medicine, The First Affiliated Hospital of Zhengzhou University, Zhengzhou, China; ^6^ School for Mental Health and Neuroscience, Maastricht University, Maastricht, Netherlands; ^7^ Department of Neurology, Maastricht University Medical Center (MUMC +), Maastricht, Netherlands; ^8^ Academic Centre for Epileptology Kempenhaeghe/MUMC+, Maastricht, Netherlands

**Keywords:** anti-GABABR encephalitis, clinical characteristics, MRI, prognosis, small cell lung cancer

## Abstract

**Introduction:**

Anti-GABABR encephalitis is a rare disease reported to be often associated with tumors. The current study aims to summarize the clinical characteristics, imaging features, treatments, outcomes and explore the potential prognosis risk factors of patients with anti-GABABR encephalitis.

**Methods:**

Patients tested positive for anti-GABABR were retrospective studied from a single medical center in China over a period of 3 years. They were followed up for a maximum period of 18 months. Clinical data were summarized and prognostic factors including demographic characteristics, laboratory tests, and neurological functions were compared between survived and deceased patients at 18 months follow-up.

**Results:**

Twenty-six patients, 10 females (38.5%) and 16 males (61.5%), diagnosed with anti-GABABR encephalitis were studied. The median age was 58 years. Of the 23 cases with complete clinical data, their main manifestations were epileptic seizures (65%), mental and behavioral abnormalities (52%), and cognitive impairment (48%). 7 (30.4%) cases had tumors: 5 small cell lung cancer (SCLC), 1 rectum adenocarcinoma (moderately differentiated) and 1 esophageal squamous cell carcinoma. MRI showed 5 (22%) cases had T2 FLAIR increased signals in cortex but with different regions affected. One of the two patients scanned for PET-CT showed hypermetabolism in the left temporal lobe region. The disease course ranged from 5 days to 3 years. 2 patients (one had esophageal carcinoma) without immunotherapy and 3 patients (one had SCLC) that did not response to immunotherapy died soon after diagnosis. 18 patients improved after immunotherapy while 3 (all had SCLC) died after relapses. The prevalence of epileptic seizures and malignancies was significantly lower in the survival group than in the deceased group at 18-months follow-up, the same as the admission mRs score. Serum fibrinogen, cerebrospinal fluid immunoglobulin G quotient, and 24-hour intrathecal synthesis rate were significantly lower in the survival groups as well.

**Conclusions:**

Cortex T2 FLAIR abnormalities were only observed in a small proportion of anti-GABABR encephalitis patients with heterogeneous MRI phenotypes. High mRS score at admission, epileptic seizures and the presence of a tumor indicated a poor prognosis, while the underlying mechanism of the later two factors should be investigated further.

## 1 Introduction

Autoimmune encephalitis is mediated by autoantibodies against different neuronal surface proteins and ion channels in the brain. Since the discovery of anti-N-methyl-D-aspartate receptor (NMDAR) encephalitis (1), many other autoantibodies targeting antigens in the brain have been identified (2, 3). Anti-gamma-aminobutyric acid type B receptor (Anti-GABABR) encephalitis was first described by Lancaster et al. in 2010 in patients with predominant limbic symptoms including seizures, mental and behavioral abnormalities, and memory loss, in the presence [mostly small-cell lung cancer (SCLC)] or absence of a tumor (4). GABABR antibodies were also found in patients with ataxia, opsoclonus-myoclonus syndrome (OMS), status epilepticus, or rapidly progressive dementia indicating that diverse clinical phenotypes may be related to this antibody (5, 6).

The presence of anti-GABABR in cerebrospinal fluid (CSF) and/or serum, mostly detected by fixed cell-based assay that make use of HEK cells over-expressing GABABR1 and GABABR2 subunits, is the key factor for diagnosis. Other assays can also confirm positive identification of anti-GABABR, such as live cell-based assay, rat brain immunohistochemistry, and/or cultured primary neurons (1, 4, 7). The sensitivity of the CSF for anti-GABABR identification was reported to be higher than serum, whereas low levels of anti-GABABR antibodies could also be identified in CSF or serum samples from patients with other diseases, which means that the interpretation of low- antibody levels is complex (4, 5, 7). Notably, a study reported the CSF titers to be higher in 5 out of 6 patients than serum, which indicated a high prevalence of intrathecal synthesis in this studied group (8). Moreover, several reports have suggested the co-existence of other auto-antibodies along with anti-GABABR. These autoantibodies include those against GAD, VGCC, CRMP5, and SOX1 (9–12).

There have been several clinical reports of anti-GABABR encephalitis in recent years; most of them, however, focused on the demographical features of the patients with inconsistent manifestations (4–7, 13, 14). In the initial report from Lancaster et al., 2010, 15 cases were reported with anti-GABABR; all presented with seizures and other symptoms related to the limbic system. 7 of them had tumors (5 had SCLC) and other autoantibodies. Höftberger et al. reported another 20 cases in 2013, 17 of which had seizures and 19 of them developed limbic encephalitis (LE). Fifty percent of the cases had SCLC. Notably, 3 of them (6%) showed other symptoms including ataxia, OMS, and status epilepticus. Van Coevorden-Hameete, et al. reported 32 cases with anti-GABABR, 66% of the patients had tumors, mostly SCLC. The most prominent symptoms of this cohort were behavioral abnormalities (97%), and seizures (90%). Strikingly, 4 of the 32 cases (12.5%) had rapidly progressive dementia. Thus, the clinical phenotypes of anti-GABABR encephalitis are rather diverse though predominantly presenting as limbic encephalitis, associated with SCLC. Studies from China reported similar conclusions as mentioned above, while whole-body PET-CT scans were more often used and showed hypermetabolism in the limbic system of the affected patients (14, 15). Additionally, several studies conducted follow-up assessments on patients to explore their prognosis, with a particular focus on mortality and associated factors (5, 6, 15–17). The main conclusions were that age of onset correlates with the outcomes but survival rates were similar between patients with SCLC and those without.

In the current study, we analyzed a Chinese cohort of 26 patients with anti-GABABR encephalitis. We especially analyzed prognostic differences among patients with and without cancer.

## 2 Methods

### 2.1 Patient selection

We retrospectively included all patients with anti-GABABR antibodies who were hospitalized at the First Affiliated Hospital of Zhengzhou University from January 2019 to April 2022. The inclusion criteria was the presence of (1) positive anti-GABABR receptor antibodies in CSF and/or serum before treatment using routinely commercial cell-based assays (Euroimmun AG, Luebeck, Germany; Simcere, Nanjing, China or MYBiotech, Xi’an, China); (2) predominant clinical symptoms consistent with autoimmune encephalitis as suggested by the international guideline (1) This study was approved by the ethical review committee of the first affiliated hospital of Zhengzhou university (2019-KY-018). And written informed consent was obtained from the individuals or legal guardians or representatives for the publication of any potentially identifiable images or data included in this article.

### 2.2 Data collection

Clinical demographic characteristics, serological indexes including inflammation and coagulation markers, CSF parameters, imaging features, and treatments were retrospectively collected via medical records. The cancer diagnosis was based on imaging and/or pathological examination. In this study, all patients with tumors underwent pathological diagnosis.

### 2.3 Treatment and prognosis evaluation

All patients received symptomatic treatment and/or immunotherapy. Immunotherapy included first-line therapy, including corticosteroids (methylprednisolone pulse therapy, sequential oral prednisone), intravenous immunoglobulin or plasma exchange, and/or second-line immunosuppressive drugs, including cyclophosphamide, and rituximab. The modified Rankin scores of early-onset and follow-up were evaluated by one experienced observer (D.L.) according to the medical records and outpatient follow-ups after discharge for a maximum of 18 months.

### 2.4 Statistical analysis

The data were statistically analyzed using SPSS25.0 (IBM Corporation, Armonk, NY, USA). The normal distribution, skewed distribution, and counting data are expressed as mean ± SD (standard deviation), median (lower quartile, upper quartile), and frequency (constituent ratio), respectively. T-test, Mann-Whitney U- test and Fisher exact test were used to compare between groups respectively. The Kaplan-Meier survival curves of the two groups were analyzed by the log-rank test. The data were all two-tailed statistics, and the significance level was set to p<0.05.

## 3 Results

### 3.1 Demographic data, clinical features, treatments and outcomes

Twenty-six patients tested positive for anti-GABABR were included. There were 10 females (38.5%) and 16 males (61.5%), with an average age of 59 years (range 16-81 years). We analyzed plasma and CSF from 22 patients, there were 9 (41%) patients positive in both plasma and CSF for GABABR autoantibodies, in 5 (23%) patients only in CSF and in 8 (36%) patients only in plasma. The dilution ranged from 1:10 to 1:1000 in plasma and from 1:3.2 to 1:1000 in CSF. In total, 85% of the patients had seizures in the whole course of the disease while 46% of the patients initially presented with seizures. Clinical data of 3 patients was (partially) unavailable. For the remaining 23 cases, 65% of the patients developed 2 or more time of seizures who were diagnosed as epileptic seizures and received anti-epileptic drugs during the course of the diseases. Other main clinical manifestations included mental and behavioral abnormalities (52%), and cognitive impairment (48%). 7 (30.4%) cases had tumors: 5 small cell lung cancer (SCLC), 1 rectum adenocarcinoma (moderately differentiated) and 1 esophageal squamous cell carcinoma. Among the described patients with a tumor, cases 5, 8, 9, 13, and 19 developed encephalitis before the tumor diagnosis, whereas cases 10 and 16 developed encephalitis after the tumor diagnosis (**Supplementary Table 1**). 21 patients were treated according to the clinical guidelines of autoimmune encephalitis, with either glucocorticoids, IVIG or plasma exchange (at least one or a combination of these). Eighteen (85.7%) patients responded well to this first-line immunotherapy, while 6 patients died during the follow-up: 3 patients (one had SCLC) that did not response to immunotherapy died soon after diagnosis and 3 (all had SCLC) died after relapses. The other 2 patients (one had esophageal carcinoma) received Ganciclovir (5mg/kg, Q12h: every 12 hours) and valproic acid (1mg/kg/hour) that had a rapidly progressive course leading to death soon after admission before immunotherapy started. The demographic data, clinical symptoms, imaging features, treatment, and outcomes of all patients with GABABR encephalitis were summarized in **Table 1**. Other antibodies and classical paraneoplastic anti neuronal antibodies were tested negative (**Supplementary Table 2**). In this study, we evaluated the correlation between the antibody titers in cerebrospinal fluid (CSF) and serum and the severity of the disease. There is a trend for a positive correlation, but it is not statistically significant (**Supplementary Figure 1**).

**Table 1:** Demographic data, clinical features, and prognosis of the 23 cases with anti-GABABR encephalitis.

Patient	Gender	Age (Year)	Initial symptoms	Tumor	Tumor diagnosis/exclusion method	Plasma Abs titers	CSF Abs titers	Head MRI (T2-flair)	Duration^1^	Immunotherapy^2^	Response to treatment^3^	Follow-up (mRS)^4^
Case 1	Female	63	Psychotic symptoms	No	CT chest,abdomen,and pelvis; abdominal ultrasound	not available	1:10	High signal in bilateral frontotemporal occipital lobe, right parietal lobe, and left insular lobe	40 days	Methylprednisolone 0.5 g, 3.5 days, decreasing	Good	0
Case 2	Female	58	Seizures^5^	No	CT chest,abdomen,and pelvis; abdominal ultrasound	not available	1:10	Hyperintensity of the left occipital cortex with swelling	20 days	IVIG 0.4mg/kg 5 days; Methylprednisolone 1g, decreasing	Moderate	1
Case 3	Male	68	Memory loss	No	CT chest; abdominal ultrasound	Negative	1:32	Increased signal in left hippocampus	10 months	IVIG 0.4mg/kgX5 days	Moderate	1
Case 4	Female	48	Seizures^5^	No	CT chest,abdomen,and pelvis; abdominal ultrasound; PET-CT	1:100	1:100	Abnormal signal in the left hippocampus	6 months	Plasma 40ml/kgX5 days; IVIG 0.4mg/kgX5 days; Methylprednisolone 1g, decreasing	Good	0
Case 5	Male	59	Decrease of speech, memory loss	Rectum adenocarcinoma	CT chest,abdomen,and pelvis; abdominal ultrasound	1:32	1:32	Bilateral frontal lobe, left temporal insula abnormal signal	12 months	IVIG 0.4mg/kgX5 days	Moderate	1
Case 6	Male	55	Seizures^5^	No	CT chest,abdomen,and pelvis; abdominal ultrasound	Negative	1:32	High signal in bilateral frontal-parietal lobes, demyelination considered	2 months	Dexamethasone 10mg X12 days later, 60mg prednisone, reduced by 10mg in each week	Good	0
Case 7	Female	69	Cognitive problems	No	CT chest,abdomen,and pelvis; abdominal ultrasound	Negative	1:100	High signal in bilateral parietal lobes, demyelination considered	5 days	No immunotherapy (Epilepsy symptom control and psychiatric symptom control treatment)	n/a	6
Case 8	Female	44	Seizures^5^	Samll cell lung cancer (SCLC)	CT chest,abdomen,and pelvis; abdominal ultrasound; pulmonary puncture biopsy	1:10	1:32	High signal observed in bilateral frontal lobes, demyelination considered	18 days	IVIG 0.4mg/kg X5 days	Moderate	2
Case 9	Male	55	Seizures^5^	SCLC	CT chest,abdomen,and pelvis; abdominal ultrasound; pulmonary puncture biopsy	1:32	1:3.2	Small ischemic foci in bilateral fronto-parietal lobes, abnormal signal in left medulla oblongata	2 months	IVIG 0.4mg/kg X5 days, 0.5g methylprednisolone decreasing	Good	6 (relapse)
Case 10	Male	62	Cognitive problems	Esophageal carcinoma	CT chest,abdomen,and pelvis; abdominal ultrasound;surgical pathological examination	Negative	1:10	High signal in bilateral frontal-parietal lobes and adjacent to both ventricles, demyelination considered	5 months	No immunotherapy(Epilepsy symptom control and psychiatric symptom control treatment)	n/a	6
Case 11	Male	60	Fever, mood changes	No	CT chest,abdomen,and pelvis; abdominal ultrasound	1:32	Negative	High signal in bilateral frontal-parietal lobes and adjacent to both lateral ventricles, demyelination considered	9 days	Methylprednisolone 1g, decreasing	Good	1
Case 12	Male	72	Psychotic symptoms	No	CT chest,abdomen,and pelvis; abdominal ultrasound	not available	1:10	High signal adjacent to both ventricles in bilateral frontal-parietal lobes, demyelination considered	16 days	Methylprednisolone 0.5g, decreasing	Good	0
Case 13	Male	55	Seizures^5^	SCLC	CT chest,abdomen,and pelvis; abdominal ultrasound; pulmonary puncture biopsy	1:32	n/a	Punctate high signal in the right frontal lobe, demyelination considered	18 days	IVIG 0.4mg/kgX5 days	Good	6 (relapse)
Case 14	Female	42	Seizures^5^	No	CT chest,abdomen,and pelvis; abdominal ultrasound	1:100	1:32	Normal	40 days	IVIG 0.4mg/kgX5 days, 0.5g methylprednisolone decreasing	Good	1
Case 15	Male	55	Seizures^5^	No	CT chest,abdomen,and pelvis; abdominal ultrasound; PET-CT	1:10	1:10	High signal in bilateral frontal lobes and the left parietal lobe, demyelination considered	35 days	IVIG 0.4mg/kgX5 days, 60mg prednisolone (oral)	No	6
Case 16	Male	73	Seizures^5^	SCLC	CT chest,abdomen,and pelvis; abdominal ultrasound; pulmonary puncture biopsy	1:100	Negative	High signal in bilateral frontal-parietal lobes, demyelination considered	5 months	IVIG 0.4mg/kgX5 days, hormone 40mg methylprednisolone	No	6
Case 17	Female	65	Seizures^5^	No	CT chest,abdomen,and pelvis; abdominal ultrasound	1:1000	1:1000	Abnormal regional cortical signal in bilateral temporal lobes and left hippocampus; Demyelination	9 days	IVIG 0.4mg/kgX5 days	No	6
Case 18	Female	63	Fever, communication disabilities	No	CT chest; abdominal ultrasound	1:10	Negative	High signal in bilateral frontal lobes and adjacent to both ventricles, demyelination considered	1 month	Plasma 40ml/kgX5 days, methylprednisolone 120mg	Good	0
Case 19	Male	67	Seizures^5^	SCLC	CT chest,abdomen,and pelvis; abdominal ultrasound; transbronchial lung biopsy	1:100	1:100	High signal observed in bilateral frontal lobes, demyelination considered	2 months	Methylprednisolone 1g, decreasing, IVIG 0.4mg/kgX5 days, plasma 40ml/kgX5 days	Moderate	6 (relapse)
Case 20	Male	16	Mental and behavioral abnormalities	No	CT chest,abdomen,and pelvis; abdominal ultrasound	1:32	Negative	n/a	3 months	Prednisone 60mg, Mycophenolate Mofetil 0.25g, 2 times/day	Good	0
Case 21	Male	59	Psychotic symptoms	No	CT chest,abdomen,and pelvis; abdominal ultrasound	1:32	1:32	High signal adjacent to both lateral ventricles, demyelination considered	36 months	IVIG 0.4mg/kgX5 days	Good	0
Case 22	Male	81	Weakness of limbs, speech impairment	No	CT chest,abdomen,and pelvis; abdominal ultrasound	1:100	Negative	High signal in bilateral frontal-parietal lobes and adjacent to both lateral ventricles, demyelination considered	17 days	Methylprednisolone 120mg	Good	0
Case 23	Female	46	Mental and behavioral abnormalities	No	CT chest,abdomen,and pelvis; abdominal ultrasound	1:32	Negative	Normal	33 days	Methylprednisolone 120mg	Good	0
Case 24	Female	68	Fever, mood changes	n/a	CT chest,abdomen,and pelvis	1:10	Negative	n/a	7 days	n/a	not available	n/a
Case 25	Male	72	Seizures^5^	n/a	CT chest,abdomen,and pelvis	1:32	Negative	n/a	1 month	n/a	not available	n/a
Case 26	Male	63	Cognitive problems	n/a	CT chest,abdomen,and pelvis	Negative	1:32	High signal in bilateral frontal-parietal lobes and adjacent to both lateral ventricles, demyelination considered	15 days	n/a	not available	n/a

^1^ Duration: the course from disease onset to discharge.

^2^ Immunotherapy: Methylprednisolone decreasing strategies were different depending on the initial dose.

^3^ Response to treatment: A good response to treatment is indicated if the mRS score is 0 within 3 months without accompanying behavioral or psychotic symptoms. A moderate response is observed if there are residual symptoms persisting beyond 3 months but showing improvement compared to the starting point of immunotherapy. A no response is indicated if the mRS score becomes higher than the mRS score at admission.

^4^ Follow-up (mRS): the follow-up was for 18 months (except one patient who was followed up for 12 months) or up to the clinical death of the patient.

^5^ To diagnose epilepsy in patients, both clinical symptoms (seizures) and electroencephalograms (EEGs) abnormalities were taken into account.

n/a, not available.

### 3.2 Imaging features

**Figure 1:**
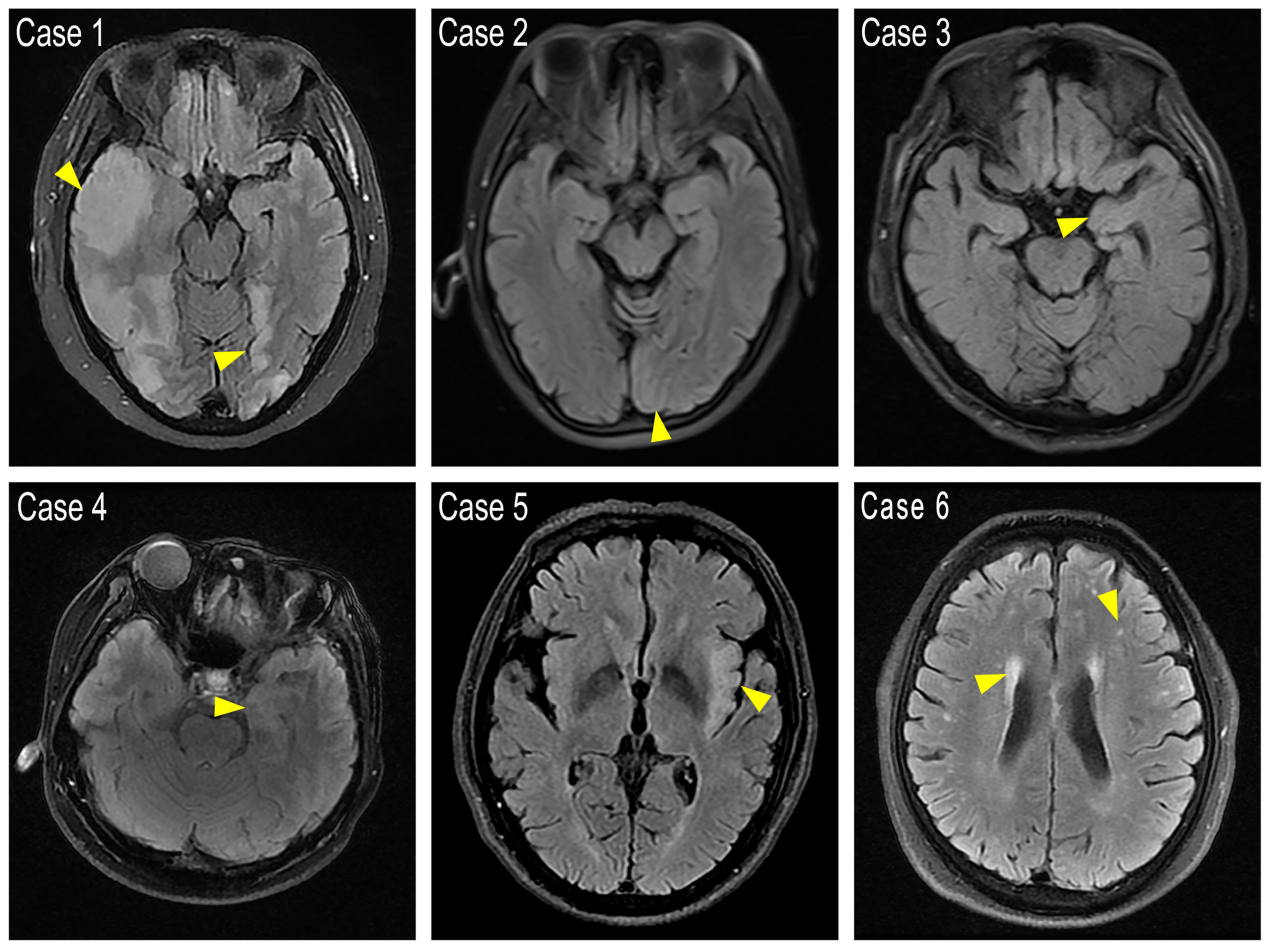
FLAIR images showing cortex abnormal signals. Case 1: Abnormal high signal was found in the bilateral frontotemporal occipital lobe, right parietal lobe, and left insular lobe with main involvement of the cerebral cortex. The boundary between gray and white matter is unclear. Case 2: A hyper-intensity in the left occipital cortex with swelling of the gyrus. Case 3 and Case 4: Left hippocampus showed slight hyper-intensity. Case 5: Bilateral frontal lobes, left temporal insular lobes present with abnormal signals. Case 6: Representative demyelination case with bi-ventricular zone, parietal temporal lobe with matter dots-like hyper signals. Yellow arrows point to the abnormal high intensity areas.

**Figure 2:**
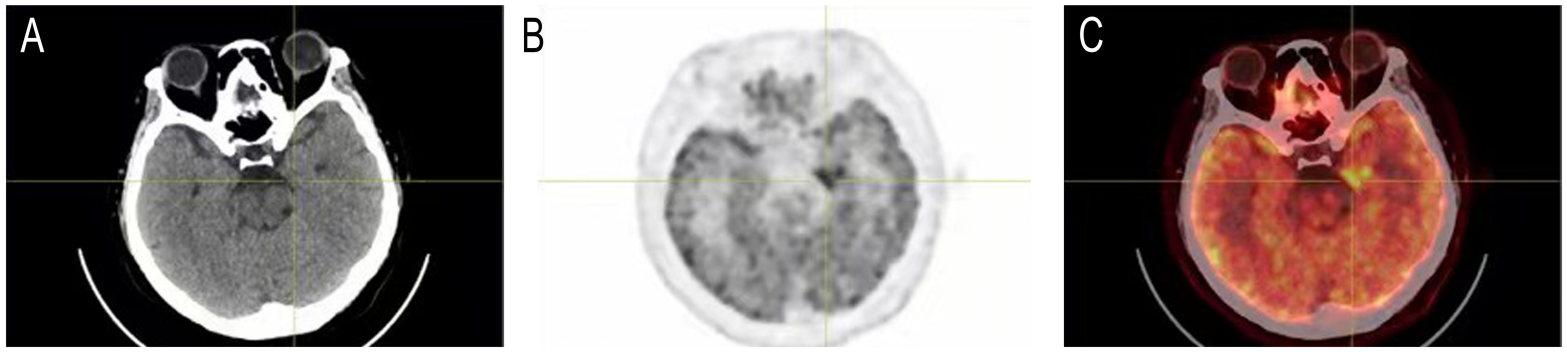
Head PET-CT of case 4. **(A)** CT imaging. **(B)** PET imaging. **(C)** Merged of CT and PET imaging showed concentrated radioactive distribution in the medial temporal lobe of the left side.

5 out of the 23 cases with available MRI showed T2 FLAIR abnormalities that affected rather different lobes uni- or bi-laterally, including frontal and temporal lobe, hippocampus or occipital cortex(MRIs of the 5 cases with T2 FLAIR abnormalities and 1 representative case with demyelination were shown in **Figure 1**). 2 patients with tumors were scanned by PET-CT to detect metastasis, and 1 showed brain metabolism abnormality related to the MRI changes (Case 4, PET-CT shown in **Figure 2**). It is of concern whether patients developed a malignancy during the 18-months follow-up. All patients in the study underwent a CT scan in the first 3 months of their follow-ups where no additional malignancies were detected. No extra patients presented symptoms related to malignancies after the first 3 months follow-up, thus they did not undergo radiological examinations further.

### 3.3 Prognosis and risk factors: comparison between the survival and the deceased groups

In the current study, 23 patients were followed up for 18 months after discharged. As mentioned above, in total 8 patients died during this period, of whom 3 (including 2 who had not received immunotherapy) within 1 month, 3 between 1-6 months and 2 patients between 12-18 months. Throughout the treatment and follow-up period, it was observed that cases 7, 9, and 19 died due to multi-organ failure after experiencing epileptic seizures. Cases 7, 15, and 17 died following respiratory failure. During the tumor treatment, cases 10 and 16 experienced a decline in immune function, leading to infections and subsequent multi-organ failure resulting in death. Analyzing the early deaths of patients like cases 7 (5 days) and 17 (9 days), it is evident that immediate onset of respiratory failure or multi-organ failure after epileptic seizures warrants high vigilance for potential early death in these patients. To explore prognostic factors, we compared the demographic data between the survival and the deceased groups. More patients in the deceased group had seizures (7 out of 8) compared to the patients who survived (8 out of 15) (Fisher exact test, p=0.04). Also, more patients had an underlying malignancy in the deceased group (5 out of 8) compared to the patients who survived (2 out of 15) (Fisher exact test, p=0.03). We did not find differences in other parameters of the demographic and clinical data (**Table 2**, **Supplementary Figure 2**, **Supplementary Table 1**).

**Table 2:** Demographic data and clinical manifestations of patients with anti-GABABR antibodies, grouped by survival status.

	Total	Deceased group	Survival group	p-value**
Patient	23	8	15	
Male, (n, %)	14 (60.8%)	6 (75%)	8 (53.3%)	0.29
Age (year)	59(55-72.6)	63.5 (55-68.5)	59 (46-63)	0.27
Main symptoms				
Epileptic seizures	15 (65.2%)	7 (87.5%)	8 (53.3%)	0.04
Mental and behavioral changes	12 (52.2%)	3 (37.5%)	9 (60%)	0.28
Cognition decline	11 (47.8%)	3 (37.5%)	8 (53.3%)	0.68
Speech disturbances	5 (21.7%)	1 (12.5%)	4 (26.7%)	0.62
Limb movement disorders	3 (13%)	1 (12.5%)	2 (13.3%)	1
Consciousness alteration	4 (17.4%)	2 (40%)	2 (13.3%)	0.59
Comorbidities				
Hypertension	1 (4.34%)	1 (12.5%)	0	0.35
Diabetes	5 (21.74%)	2 (40%)	3 (20%)	1
Trauma	2 (8.70%)	1 (12.5%)	1 (6.7%)	1
CTD*	5 (21.74%)	2 (40%)	3 (20%)	1
With tumor	7 (30.44%)	5 (62.5%)	2 (13.3%)	0.03

*CTD, Connective tissue disease.

** Mann-Whitney U- test were used to compare the age distribution difference between the groups and fisher-exact test was used for the remaining comparisons (frequency).

In the current study, both available blood inflammation and coagulation biomarkers were collected and compared between the survival and deceased groups. There were significant differences in serum fibrinogen, cerebrospinal fluid IgG quotient, and 24-hour intrathecal synthesis rate between the two groups (p<0.05) (**Table 3**). Considering that both fibrinogen and IgG intrathecal synthesis are in general related to tumors, the three parameters (fibrinogen, IgG quotient, 24-hour intrathecal synthesis) were also compared between the group with tumor and the group without tumor. Differences were significant for fibrinogen (P=0.009) and 24-hour intrathecal synthesis rate (P=0.03), which indicates that the presence of the tumor might be the key factor related to the death.

**Table 3:** Serology and cerebrospinal fluid parameters of patients with anti-GABABR antibodies, grouped by survival status.

	Deceased group	Survivors group	p-value*
Blood inflammation biomarkers			
ESR (mm/hour)	16.81 (± 9.30)	11.49 (± 8.75)	0.19
C3 (mg/dL)	1.09 (1.09-1.24)	1.09 (1.09-1.24)	0.17
C4 (mg/dL)	0.26 (0.26-0.37)	0.26 (0.24-0.29)	0.09
C-reactive protein (mg/mL)	3.15 (2.24-19.33)	2.44 (1.00-6.3)	0.27
PCT (ng/mL)	0.03 (± 0.02)	0.30 (0.30-0.30)	0.88
Blood coagulation markers			
D-dimer (μg/mL)	0.55 (0.19-1.78)	0.30 (0.19-0.46)	0.33
Fibrinogen (mg/mL)	3.67 (± 0.76)	2.85 (± 0.89)	0.04
Fibrin Degradation Products (ug/mL)	2.83 (2.5-9.63)	2.50 (2.50-2.50)	0.36
Prothrombin Time (s)	10.90 (9.65-12.20)	10.70 (10.40-11.50)	0.88
Activated Partial Thromboplastin Time (s)	29.16 (± 2.06)	27.03 (± 4.04)	0.18
CSF parameters			
WBC	4(2-36.50)	2 (2-4)	0.10
Lymphocytes (%)	76% (± 10.80%)	69.07% (± 8.51%)	0.11
Cerebrospinal fluid protein (mg/L)	408.85 (287.41-644.98)	279 (251.4-412.40)	0.08
Cerebrospinal fluid albumin (mg/dl)	22.3 (19.64-28.08)	22.3 (15.37-25.66)	0.88
IgG quotient	5.38 (3.94-13.75)	3.87 (3.02-4.06)	0.047
Albumin quotient	5.68 (5.68-6.80)	5.38 (3.10-6.51)	0.33
Presence of oligoclonal bands **	2 (25%)	3 (2%)	1
24-hour intrathecal synthesis rate (mg/24 hours) *	14.29 (5.76-42.43)	5.50 (1.72-6.43)	0.047

*The normal distribution, skewed distribution, and counting data are expressed as mean ± SD (standard deviation), median (lower quartile, upper quartile), and frequency (constituent ratio), respectively. T-test, and Mann-Whitney U- test and Fisher exact test were used to compare between groups respectively.

**In this study, the reagent used for detecting oligoclonal bands is a cerebrospinal fluid immunoglobulin G monoclonal band detection kit (enzyme-linked immunofixation electrophoresis method, Sebia company.

***Using the Tourtellotte revised formula: 24-hour intrathecal synthesis rate = [(IgG_CSF - IgG_S/K1) - (Alb_CSF - Alb_S/K2) × (IgG_S/Alb_S) × 0.43] × 5; K1=normal person(serumIgG/csfIgG); K2=normal person(serumAlb/csfAlb).

**Figure 3:**
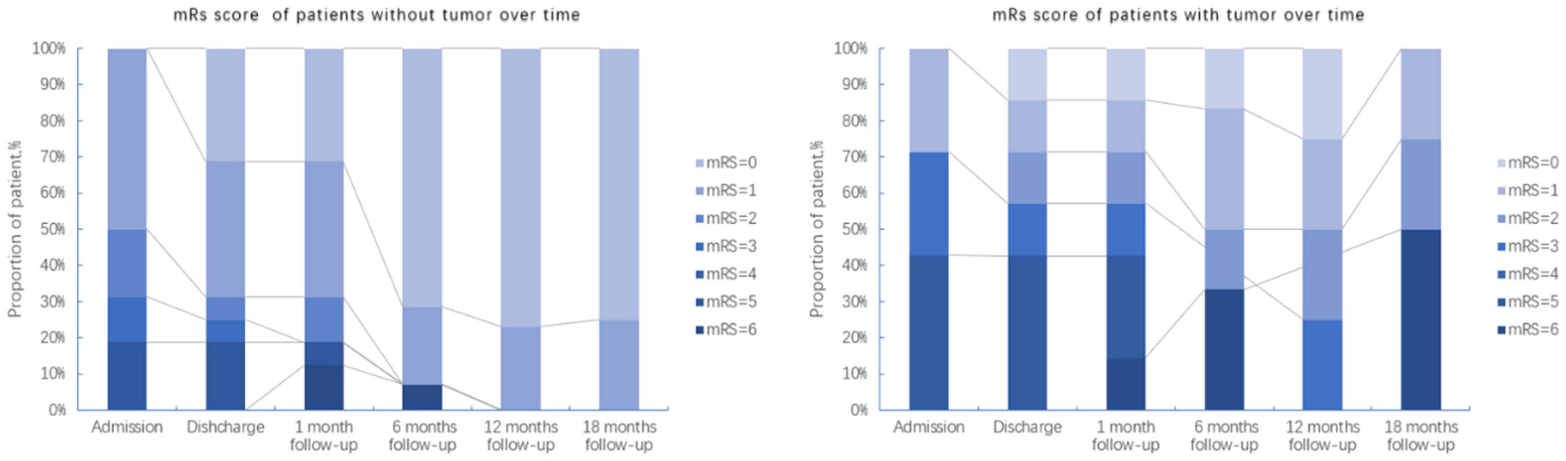
Follow-up of mRS scores from patients with and without tumors over a maximum period of 18 months.

The mRS scores of patients at each stage (Admission, Discharge, 1-, 6-, 12- and 18-months follow-ups) were analyzed to evaluate the prognosis of the disease. Overall, 6 out of the 8 patients from the deceased group had mRS scores up to 5 at admission compared to no patients from the survival group with such high mRS score at admission (6/8 vs 0/15, P=0.0003, Fisher exact test, **Supplementary Figure 3**). Thus, a high mRS score at admission strongly related to a bad prognosis. Considering that a tumor was a key feature that was present in one-third of the cohort and significantly related to mortality, we represented the changes of mRS scores over time of patients with tumor and without tumor separately (**Figure 3**, **Supplementary Figure 3**).

**Figure 4:**
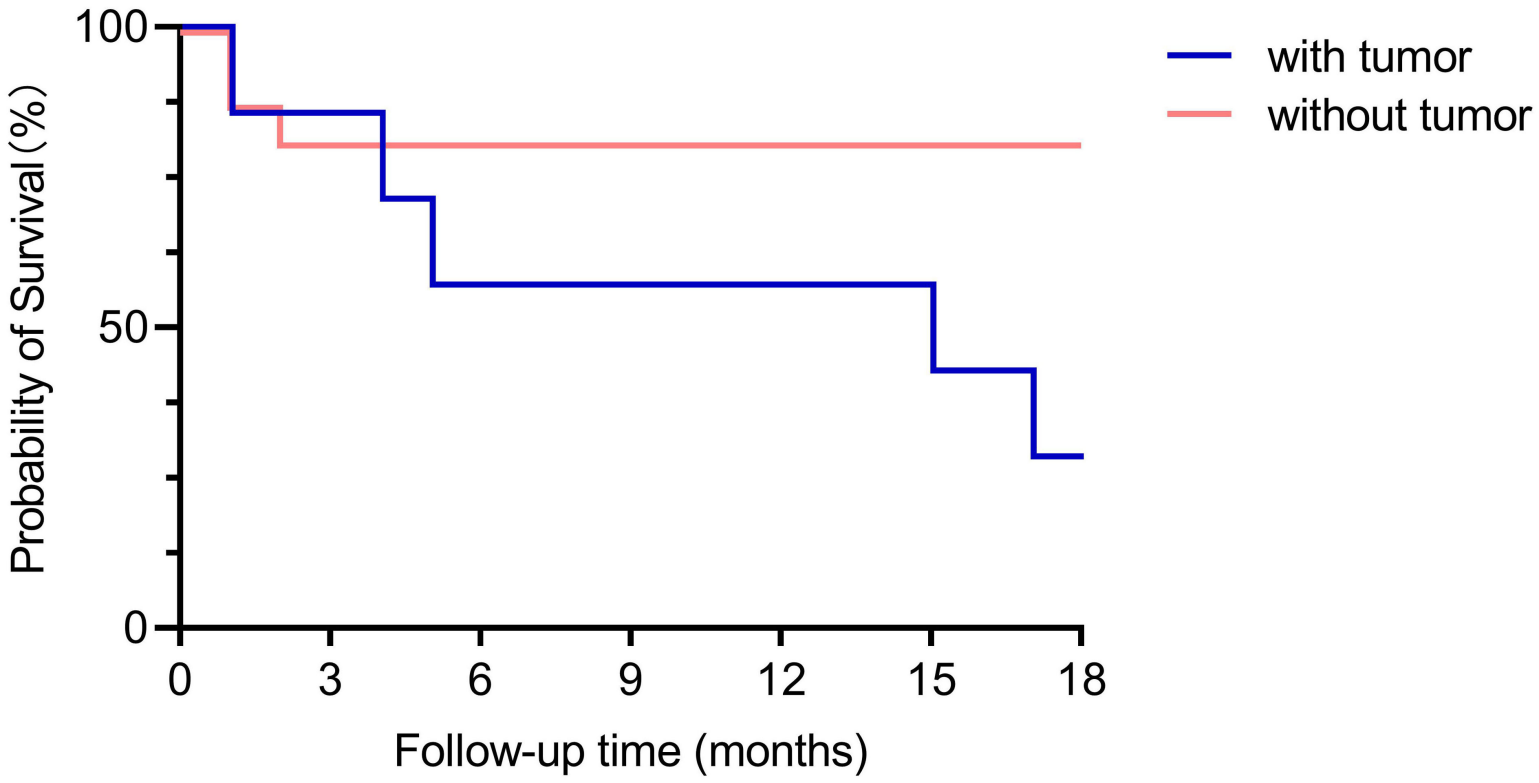
Comparison of survival curves between the groups with and without tumor. With a maximum follow-up of 18-months (except one case which was followed up for 12 months), the survival curves between the groups with tumor and without tumor were significant different (Log-rank (Mantel-Cox) test, P=0.028).

In general, there was no obvious difference of mRS scores at admission (between the groups with and without tumor). However, over a period of 18 months after discharge, the majority of patients without tumors recovered, only 2 of the 7 patients with tumors were alive, but still with neurological sequelae. This is also reflected in the survival curves of patients with and without tumors (**Figure 4**), which were significantly different in a Kaplan-Meier analysis (Log-rank (Mantel-Cox) test, P=0.028).

## 4 Discussion

In our study in 26 patients with GABABR encephalitis we found that seizures were the most common symptom, with a high comorbidity of malignancy, mostly with SCLC. We found that most patients responded well to treatment, but, strikingly, patients who did not survive, more often had seizures and an underlying malignancy than those patients who survived from anti-GABABR encephalitis.

GABABR is widely distributed in the central nervous system and is highly expressed in the hippocampus, thalamus, and cerebellum. GABABR is a receptor for an inhibitory neurotransmitter, with an essential function in the brain, not only influencing learning, memory, and cognition but also movement and balance (18, 19). Frequent seizures can occur in GABABR gene knockout mice (20, 21). In line with this, early severe seizures are an important clinical manifestation of anti-GABABR encephalitis, which are usually resistant to anti-epileptic drugs and can be relieved only after immunotherapy (4, 9, 15). We found in our cohort that 85% cases had seizures. This finding is in line with other studies, which found that around 90% of the cases had seizures (4–6, 16, 22, 23). Although Limbic symptoms (seizures, cognitive impairment, and psychiatric abnormalities) are the main features of GABABR encephalitis, we reported slightly low prevalence (65.2%) of seizures compared to previous reports. Lancaster et al. in 2009 first reported GABABR encephalitis in 15 patients, all with seizures and Hoftberger et al. in 2013 reported 20 GABABR encephalitis patients, 90% of them had seizures, mostly as part of the limbic syndromes while 1 case (5%) presented only with status epilepticus. In 2019, Van Coevorden-Hameete et al. showed that 90% of patients have epilepsy while others present with progressive dementia without seizures in cohort with 32 patients (4–6), similar findings were reported in other studies latterly (8, 13–15).This is in line with our deceased group, of which 87.5% had seizures but differs from the survival group of which 53.3% had seizures. We also noticed that the survival group showed a shorter diagnosis time and received timelier immunotherapy, which might prevent the development of seizures in these cases. It was found that the presence of epileptic seizures was related to high mortality, which might indicate that these patients suffered from more severe brain dysfunctions, with the possible benefit of early immunotherapy. Another factor that strongly associated with the clinical prognosis was the mRS score at admission, which also points towards the importance of early diagnosis and treatment.

MRI is valuable in the diagnosis of anti-GABABR encephalitis but the abnormalities found can be rather diverse. Previous MRI studies found up to 45% of patients with anti-GABABR encephalitis with pathological changes in the medial temporal lobe, uni- or bilaterally (3). We found such changes in only 21.7% of our patients, with diverse MRI characteristics that could be covered by previous reported types (4, 14, 16). Based on the MRI results of Case 1, the patient may have GABAAR antibodies rather than GABABR antibodies (24), we retested the sample and confirmed that GABAB antibodies were positive, further expanding the MRI abnormalities phenotype of GABABR encephalitis (**Supplementary Figure 4**). It is worth mentioning that whole body PET-CT would at the same time provide valuable information of possibly abnormal brain function for anti-GABABR encephalitis patients, although normally the direct purpose of using PET-CT was to scan for the occurrence of cancer or metastasis. In our study, 1 of the 2 patients who underwent PET-CT scans showed an increase of metabolism in the temporal lobe, which is consistent with the previously reported studies (14, 15).

Anti-GABABR encephalitis had two significant features as found in the current study: a relatively high mortality (34.8%) within 18 months and a fairly strong, though not unequivocal, association with malignant tumors, especially with SCLC. In previous reports, the mortality rate of anti-GABABR encephalitis was about 22%~45% (4, 5, 22, 25) Two studies indicated that the prevalence of tumors is between 33.3% to up to 50% (17, 23, 25). In our study, the proportion of cancer patients was 30.44%, so slightly lower than in previous studies. It has been reported previously that the recurrence rate of GABABR encephalitis is 9% (1/11) (26),and in the relapsed patient there were no malignancies detected. This is different from the findings in our study, We found that 13% (3/23) of patients relapsed, and all had tumors. Obviously, a malignant tumor is an ominous factor for survival (20). Here we report that the long-term prognosis of patients with tumors was worse than without a tumor. A possible explanation regarding the worse prognosis compared to patients without tumors is that the presence of a tumor continuously triggers the autoimmune reaction that causes the symptoms and thus impairs functional recovery. This indicates that management of the tumor probably is also crucial not only for survival, but also for the long-term prognosis of the course of the encephalitis.

Besides antibody detection, previous studies (4, 16, 22, 23, 26) also focused on other laboratory findings such as cerebrospinal fluid (CSF) pressure, cell count, and protein levels, as well as oligoclonal bands (OCBs) and intrathecal synthesis. In previous studies, the occurrence rate of OCBs in GABABR encephalitis varied. In the first GABABR encephalitis study from Lancaster et al. in 2009, 25% (4 out of 12) patients had OCBs in CSF (4), this is similar with our single-center study data which revealed that only 21.7% (5/23) showed positive results of OCBs (**Supplementary Table 1**). The study by Blinder & Lewerenz (27) found that out of 130 GABABR encephalitis cases, only 19 had OCB (oligoclonal bands) data available and around 75% were positive for OCBs. The high percentage of positive OCBs in these 19 cases might not give a true picture of how common they are in the whole group of 130 cases, because the number of cases with data was too small. In antibody-positive autoimmune encephalitis, cerebrospinal fluid pleocytosis and elevated protein levels are frequently detected, and similar findings may also be observed in autoimmune encephalopathy.

The increase of the CSF IgG quotient and 24-hour intrathecal synthesis rate is characteristic of demyelinating diseases of the central nervous system such as multiple sclerosis (5, 28–32). Interestingly, we also found higher levels of these parameters in patients who did not survive, coinciding with the patients with tumors. Several studies reported that patients with paraneoplastic neurological syndromes (with anti-Hu antibodies) had robust CNS involvement if they had intrathecal synthesis (33–35). In this study, we coincidentally found that the IgG intrathecal levels were high in GABABR encephalitis patients with tumors (mostly deceased), which triggers our discussion on the relationship between intrathecal synthesis and the existence of tumors. Our results may indicate the presence of a process of mature auto-reactive plasma cells which have migrated to the brain/CSF, possibly triggered by tumors (36). Fibrinogen testing is used to evaluate and monitor if people have symptoms like excessive bleeding or abnormal blockages in veins or arteries (37), and recent studies have shown that there is a relationship between the increase of fibrinogen level and the destruction of the blood-brain barrier (38–40). The level of fibrinogen in the tumor group was significantly higher than that in the group without tumor. Together, a possibility is that a hypercoagulable state from cases with tumors (similar to Trusso syndrome) damaged the blood-brain barrier in early disease stage thus allowing an increase of intrathecal synthesis of immunoglobulins, which leads to treatment resistance or poor prognosis.

In this study, we also observed that some patients died during follow-up, most of them in the early stages of the follow-up period, which is consistent with the findings of Lamblin et al. (41). We have re-examined our data and found the following potential reasons for this observation: Firstly, the number of patients in our study is relatively small, which may not provide a statistically significant representation of the expected trend; secondly, the follow-up period each 6 months might not be long enough to observe an increase in the number of deceased patients.

Our study has several shortcomings: Firstly, although the inclusion cohort was relatively larger than many other case series studies, it is still small for prognostic predictor analysis thus only correlation analysis was done. Secondly, as a retrospective study, recall biases might exist. Thirdly, the assessment of neurological dysfunction only by the modified Rankin scale has its limitation which may not reflect patients’ specific outcomes and quality of life since it focuses mostly on motor symptoms.

Although retrospective studies on anti-GABABR encephalitis exist, most are multi-center studies. Our study, based on single-center data, ensures standardized care and increases data reliability. Additionally, we explore the heterogeneity of MRI phenotypes, offering a new perspective. We also identified factors associated with poor prognosis, such as high mRS score, epileptic seizures, and the presence of tumors, which help in early identification of high-risk patients and optimizing clinical interventions. While the sample size is comparable to other studies, the 26 cases from a single center provide valuable data for researching this rare disease.

## 5 Conclusion

The prominent clinical manifestations of patients with anti-GABABR encephalitis are seizures, mental and behavioral changes, and cognitive impairment. Cortex T2 FLAIR abnormalities can only be seen in a small proportion of the patients with heterogeneous MRI phenotypes. Epileptic seizures and the presence of tumors indicated a poor prognosis, while the underlying mechanism should be investigated further.

## Data Availability

The original contributions presented in the study are included in the article/**Supplementary Material**. Further inquiries can be directed to the corresponding authors.
