# Supplementary material for: Anti-GABAB receptor encephalitis: clinical and laboratory characteristics, imaging, treatments and prognosis
Source: Front Immunol. 2024 Oct 9;15:1442733. doi: 10.3389/fimmu.2024.1442733 (PMC11496097; doi:10.3389/fimmu.2024.1442733)
Supplement: Supplementary file 1 [file DataSheet1.docx]

**Supplementary Figure 1:**


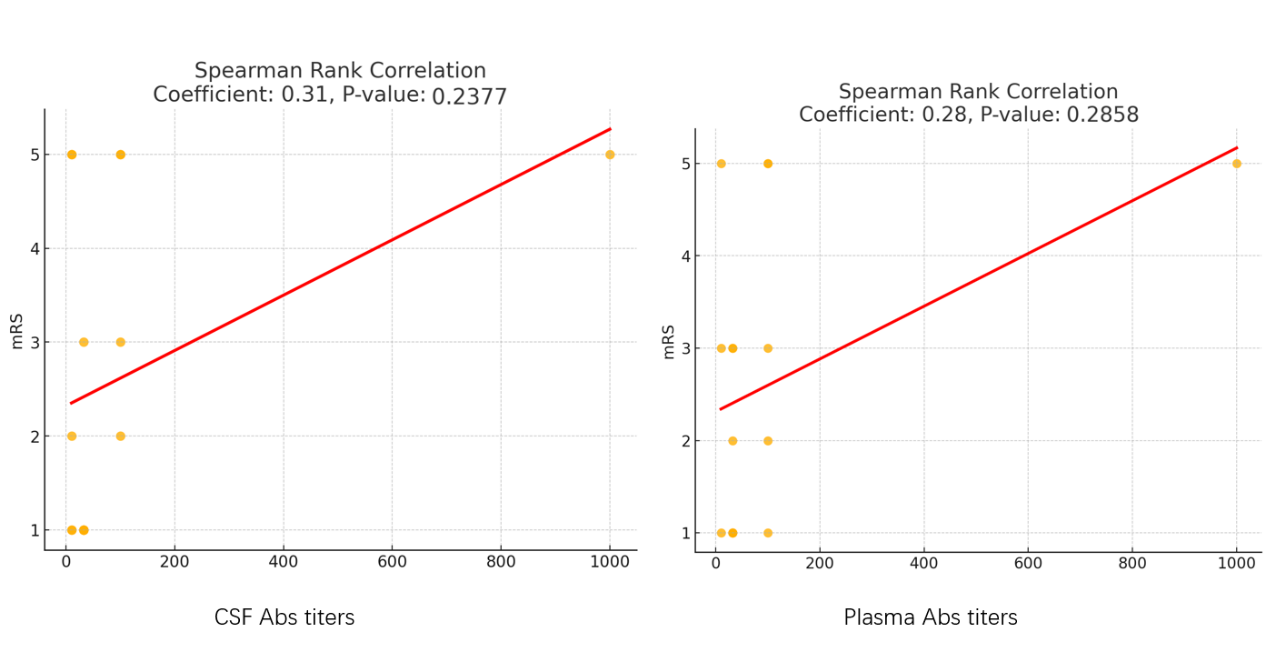


**Supplementary Figure 1.** This graph shows the correlation between antibody titers (Abs titers) from the first lumbar puncture (Cerebrospinal fluid, CSF) or blood drawn and the clinical severity of every individual. Patient CSF samples are screened for Anti-GABABR autoantibodies after being diluted to 1:1 and 1:3.2. If staining is observed at both dilutions, the laboratory will continue to dilute the sample with a dilution factor of 10 (1:10 and 1:32; 1:100 and 1:320； etc. ) until the staining is no longer visible. The Abs titer refers to the highest dilution at which recognizable staining is still observed. For plasma samples, the initial dilutions are 1:10 and 1:32, and the rest dilution steps are the same. The clinical severity was measured by the Modified Rankin Scale (mRs) score from 0 to 6. No significant correlations were found between the Abs titers in CSF or plasma with the mRs score.

**Supplementary Figure 2:**


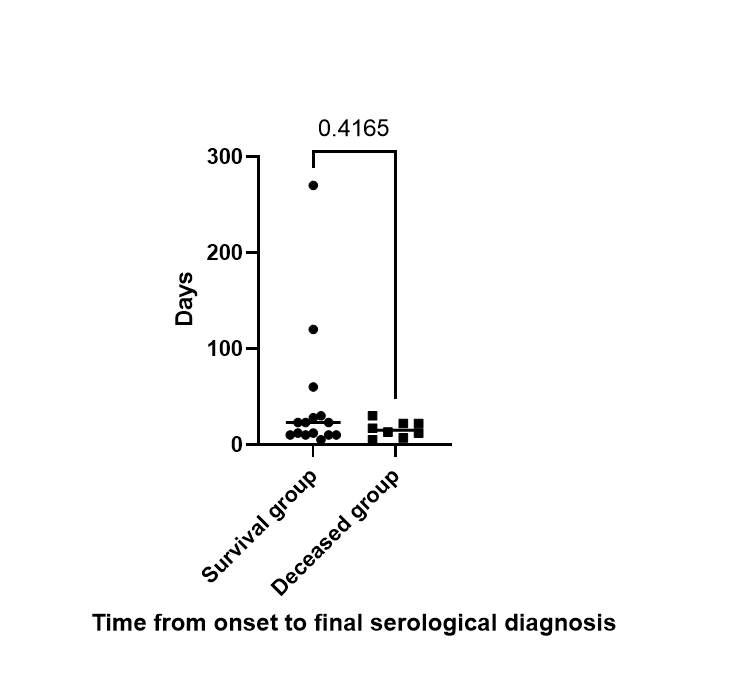


**Supplementary figure 2: Time difference from onset to final diagnosis in the survival and deceased group.** The average time from onset to final diagnosis of the survival group was 23 days (ranged from 5 to 270 days), compared to an average time from onset to final diagnosis of the deceased group of 15 days (ranged from 5 to 30 days). There were no statistically significant differences between the groups (p=0.4165).

**Supplementary Figure 3:**

**
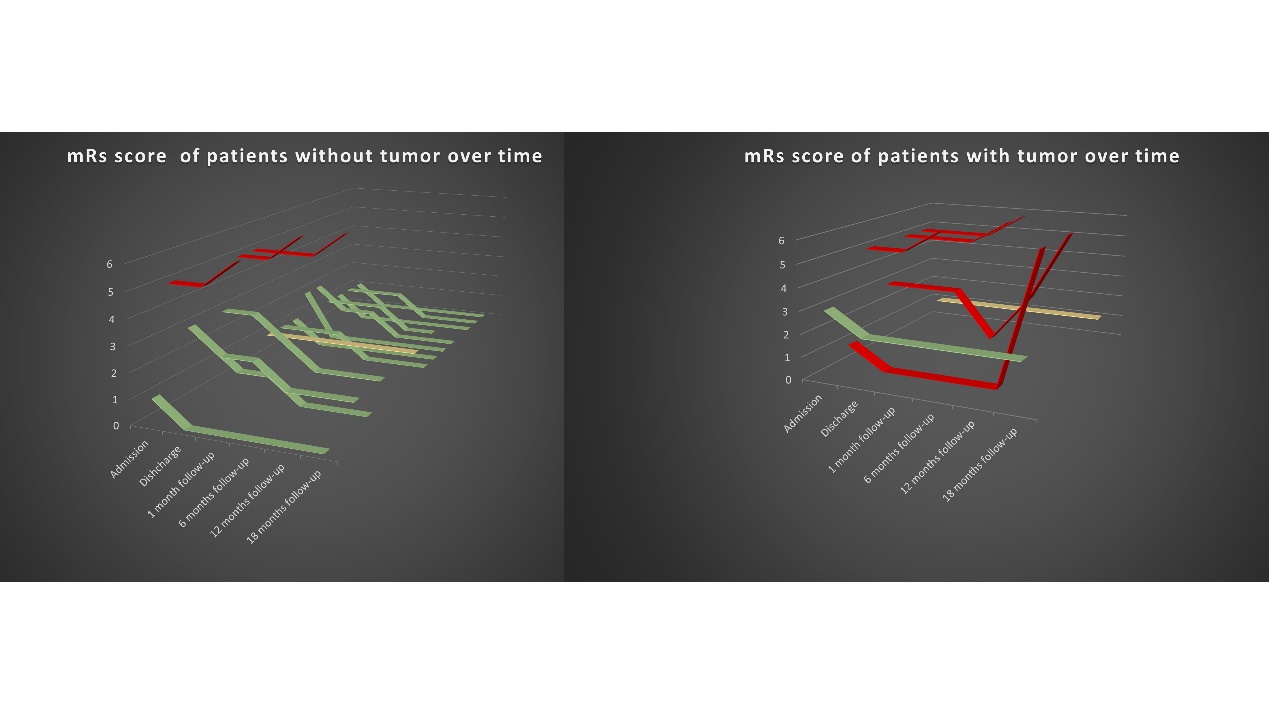
**

**Supplementary Figure 3: Follow-up of mRS score from patients with and without tumors over a maximum period of 18 months**. Each color stripe represents the change of mRS score of each case over time. Cases with an increase in mRS score (indicating worsening condition) are labeled in red, while cases with a decrease in mRS score (indicating improvement) are labeled in green. Cases showing no change in the mRS score are labeled in yellow.

**Supplementary Figure 4:**


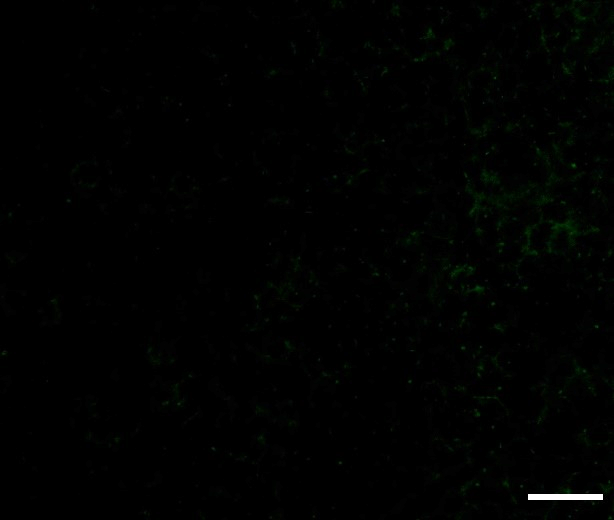

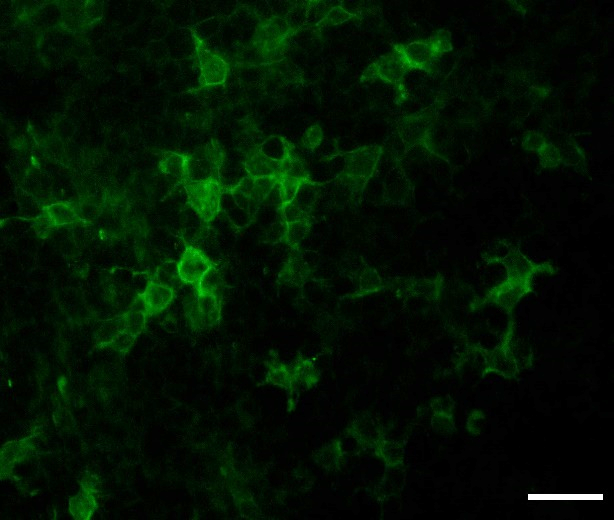


**Supplementary Figure 4.** Cerebrospinal fluid (CSF) sample from case 1 was re-tested for anti-GABABR and anti-GABAAR autoantibodies respectively using a commercial cell-based assay following the manufacturer’s instructions（Guangzhou Weimi Bio-Tech Co.,Ltd， Gunagzhou, China. It was found positive for anti-GABABR (left, green) but negative for anti-GABAAR autoantibodies (Right, green). Scale bar=50um.
